# A Taxon-Specific and High-Throughput Method for Measuring Ligand Binding to Nicotinic Acetylcholine Receptors

**DOI:** 10.3390/toxins11100600

**Published:** 2019-10-16

**Authors:** Christina N. Zdenek, Richard J. Harris, Sanjaya Kuruppu, Nicholas J. Youngman, James S. Dobson, Jordan Debono, Muzaffar Khan, Ian Smith, Mike Yarski, David Harrich, Charlotte Sweeney, Nathan Dunstan, Luke Allen, Bryan G. Fry

**Affiliations:** 1 Venom Evolution Lab, School of Biological Sciences, The University of Queensland, St. Lucia, QLD 4072, Australia; christinazdenek@gmail.com (C.N.Z.); rharris2727@googlemail.com (R.J.H.); n.youngman@uq.edu.au (N.J.Y.); j.dobson@uq.edu.au (J.S.D.); jordan_debono@hotmail.com (J.D.); 2 Department of Biochemistry & Molecular Biology, Biomedicine Discovery Institute, Monash University, Clayton, VIC 3800, Australia; sanjaya.kuruppu@monash.edu (S.K.); ian.smith@monash.edu (I.S.); 3 Institute of Biology, Leiden University (IBL), Sylvius Laboratory, Sylviusweg 72, 2333 BE Leiden, The Netherlands; m.a.khan@biology.leidenuniv.nl; 4 Millennium Science, 4 Miles Street Mulgrave, VIC 3170, Australia; myarski@mscience.com.au; 5 QIMR Berghofer, Royal Brisbane Hospital QLD 4029, Australia; David.Harrich@qimrberghofer.edu.au; 6 Translational Research Institute, University of Queensland, QLD 4072, Australia; csweeney@vaxxas.com; 7 Venom Supplies Pty Ltd., Stonewell Rd, Tanunda, SA 5352, Australialuke@venomsupplies.com (L.A.)

**Keywords:** venom, ligand, evolution, biosensor, nicotinic acetylcholine receptor

## Abstract

The binding of compounds to nicotinic acetylcholine receptors is of great interest in biomedical research. However, progress in this area is hampered by the lack of a high-throughput, cost-effective, and taxonomically flexible platform. Current methods are low-throughput, consume large quantities of sample, or are taxonomically limited in which targets can be tested. We describe a novel assay which utilizes a label-free bio-layer interferometry technology, in combination with adapted mimotope peptides, in order to measure ligand binding to the orthosteric site of nicotinic acetylcholine receptor alpha-subunits of diverse organisms. We validated the method by testing the evolutionary patterns of a generalist feeding species (*Acanthophis antarcticus*), a fish specialist species (*Aipysurus laevis*), and a snake specialist species (*Ophiophagus hannah*) for comparative binding to the orthosteric site of fish, amphibian, lizard, snake, bird, marsupial, and rodent alpha-1 nicotinic acetylcholine receptors. Binding patterns corresponded with diet, with the *Acanthophis antarcticus* not showing bias towards any particular lineage, while *Aipysurus laevis* showed selectivity for fish, and *Ophiophagus hannah* a selectivity for snake. To validate the biodiscovery potential of this method, we screened *Acanthophis antarcticus* and *Tropidolaemus wagleri* venom for binding to human alpha-1, alpha-2, alpha-3, alpha-4, alpha-5, alpha-6, alpha-7, alpha-9, and alpha-10. While *A. antarcticus* was broadly potent, *T. wagleri* showed very strong but selective binding, specifically to the alpha-1 target which would be evolutionarily selected for, as well as the alpha-5 target which is of major interest for drug design and development. Thus, we have shown that our novel method is broadly applicable for studies including evolutionary patterns of venom diversification, predicting potential neurotoxic effects in human envenomed patients, and searches for novel ligands of interest for laboratory tools and in drug design and development.

## 1. Introduction

Nicotinic acetylcholine receptors (nAChRs) are a family of ligand-gated ion channels located throughout the body for many different physiological functions. One of their most vital roles is signaling between neuronal junctions to skeletal-muscle cells [1,2,3]. At the neuromuscular junction, the binding of acetylcholine to the 14-amino acid orthosteric site (i.e., active site) of the α-1 subtype triggers the contraction of muscles [2,3]. In addition to their central role at the neuromuscular junction, nAChR α-subunits are also widely distributed in the central nervous system and therefore of pharmacological interest. There are approximately 17 characterized nAChR subunits (α-1-10, β1-4, γ, ε, σ), which structurally link to form a pentamer in homomeric or heteromeric combinations. Nicotinic acetylcholine receptors are of broad interest due to their central involvement in disease states and in mediating the paralysis of prey and human victims upon envenoming by some venomous animals.

α-nAChR subunits are targeted by toxins within venoms from a diverse range of animals including cone snails, scorpions, snakes, and spiders, as well as poisonous organisms such as dart frogs and cyanobacteria [4,5,6,7,8]. The targeting of α-1 nAChR by these toxins allows effective immobilisation of prey through the flaccid paralysis of voluntary muscles, leading to death by respiratory failure. Toxins targeting the α-1 nAChR have evolved in snake venom on at least four separate occasions: three finger toxins (e.g., α-bungarotoxin), which are widespread in the advanced snakes (Caenophidia), waglerin peptides from the *Tropidolaemus* genus of Asian pit vipers, azemiopsin peptides from the Asian viper genus *Azemiops*, and phospholipase A_2_ toxins from vipers within the *Bitis* genus [9,10,11,12]. As these four neurotoxin classes are structurally unrelated to each other and possess different protein scaffolds, they represent a remarkable functional convergence of toxins independently targeting the same neurological target (α-1 nAChRs) at the neuromuscular junction of various prey types. Furthermore, due to variations in the amino acid sequence alignments of nAChR subunits within different taxa, these neurotoxin classes represent excellent study systems for investigating how prey specific toxins evolve and, in parallel, how resistance evolves in prey and predators of venomous snakes.

A lack of high-throughput assays that measure effects upon specific-species nAChRs or receptor subtypes has been a major limitation hampering studies on the evolution of these neurotoxins, their clinical effects, and their biodiscovery. Current analytical methods to determine the binding of toxins to nAChRs are either low-throughput (in vitro skeletal muscle preparations, such as the chick biventer cervicis nerve-muscle preparation or mouse/rat phrenic nerve hemidiaphragm), cumbersome (oocyte patch-clamp systems), and/or taxonomically limited (cellular screening techniques such as Fluorescence Imaging Plate Reader (FLIPR)) [13]. In addition to limitations such as requiring animal dissections and high consumption of precious venoms and pure toxins, these assays cannot test for taxon-specific venom effects upon prey or the evolution of toxin resistance by prey. Thus, there is an unmet need for a flexible, high-throughput method that can accurately measure such biomolecular interactions. A newer, more robust biomolecular detection method of analyte-ligand binding of nAChRs is vital to overcome these hindrances. 

Short synthetic peptides (mimotopes) corresponding to the orthosteric site of nAChRs have been utilized in ligand binding studies of α-bungarotoxin to investigate their use as first aid or antivenom supplements [14,15,16,17]. These studies include the use of surface plasmon resonance (SPR), a microfluidics delivery system which clogs easily, requires experienced operators and expensive gold sensor chips, is low throughput, and has high running and maintenance costs. Consequently, the use of mimotopes to study ligand binding to nAChR orthosteric sites has remained dormant for over 15 years. Previous approaches using mimotope peptides also did not take a taxonomically diverse approach, investigating only human and rodent chimeric analogs. 

More recent research used the mollusk acetylcholine binding protein [18] and chimeric forms with human α-7 residues at the orthosteric site [19] to investigate snake venom relative potencies and the potential therapeutic usefulness as ‘decoy proteins’. In both cases, studies were hampered by the fact that snake venom nAChR targeting neurotoxins have been evolutionarily selected for the muscle-type α-1 subunit [8]. Thus, human α-7 orthosteric sequence results may be misleading for evolutionary or potential clinical effects studies and the dramatically lower affinity relative to the α-1 orthosteric site would limit the usefulness of α-7 based proteins as antivenom supplements. The mollusk acetylcholine binding protein is vastly more evolutionarily distant and therefore studies which investigated snake venom evolutionary patterns using this assay would be skewed, as was shown in one such study in which known neurotoxic snakes, such as within the *Boiga* genus, did not bind in the assay [18].

We have developed a high-throughput method which has many advantages that overcome the aforementioned limitations of current analytical approaches of nAChR binding. Our method is based upon mimotope peptides corresponding to α-nAChR subunit orthosteric sites spanning the full range of nAChR α-subunits (1–10)—not only from humans but from a wide range of model systems of potential prey types (fish, amphibian, lizard, snake, marsupial, and rodent). Combining specifically designed mimotopes with biolayer interferometry (BLI) precision enables a taxonomically robust assay to measure analyte binding to α-nAChR subunits. 

BLI is an innovative label-free, microfluidics-free, optical technique that accurately measures, in real time, the thickness of biomolecules progressively accumulating on the interaction surface of an optical-fiber coated biosensor [20]. The binding of molecules to the biosensor causes a measurable spectral shift in the waves of light being reflected through the fiber-optic biosensor, yielding quantitative, kinetic interaction information (k_on_, k_off_ and K_D_ (k_off_/k_on_)). Measuring binding strength and speed between interacting molecules of interest is essential, for example, in testing the efficacy of drugs, quantifying neurotoxic potency of venom toxins, or in determining the quality of an antibody. Unlike existing analyte-ligand binding assays of nAChRs, the versatility, high-throughput nature, taxon-specificity, and low analyte-consumption aspects of our novel assay enables the fast and accurate characterisation of the orthosteric binding profiles of analytes to nAChRs across numerous taxa.

This study investigated the use of mimotope peptides corresponding to the native orthosteric sites of a wide range of potential prey lineages in order to ascertain evolutionary patterns in snake venom neurotoxin targeting and evolution. This novel method allows for selective, taxon-specific testing of ligand binding to nAChRs for the first time. In addition, this study investigates the usefulness of this method for biodiscovery by testing for ligands that selectively bind to the full range of human α-nAChR subunits. This tool, therefore, can fast-track compound screening and purification protocols, ascertain potency rank order of neurotoxic venoms, and quantify drug binding for the progress of drug design and development. 

## 2. Results and Discussion

**Figure 1:**
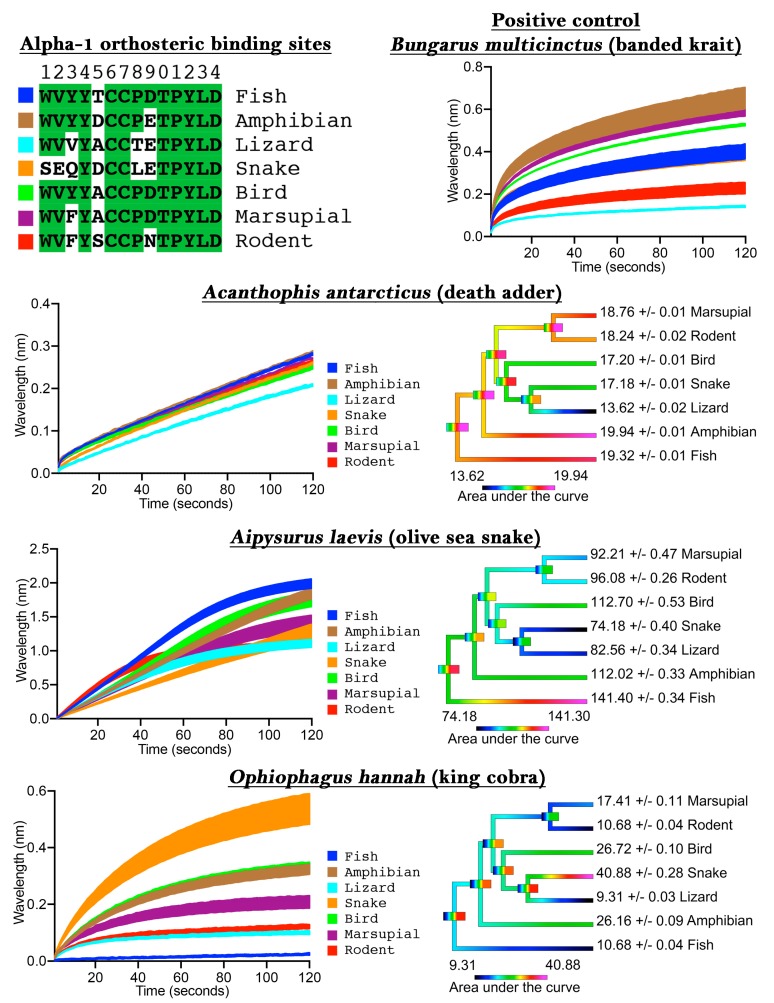
Comparisons of a generalist-feeding species (*Acanthophis antarcticus*), a fish-prey specialist (*Aipysurus laevis*), and a snake-prey specialist (*Ophiophagus hannah*). Colored rectangles next to the orthosteric site sequences (top left image) correspond to the results for the particular target in the line graphs (*B. multcinctus* image and left side images of *A. antarcticus*, *A. laevis*, and *O. hannah* panels). Green highlights show ancestral residues. Phylogenetic tree colouring is heat mapping, with lower values colored cooler while higher values are colored warmer (right side images of *A. antarcticus*, *A. laevis*, and *O. hannah* panels). Note the different scale bars for each heat map. Phylogenetic tree node bars indicate ancestral state reconstruction error range, which rapidly becomes broad due to the dynamic variation in target specificity. All values are *N* = 3 mean and SEM, with the very small error range reflective of assay precision.

To validate our assay we first tested *Bungarus multicinctus* as a positive control, as it has been shown to bind to α-1 mimotope peptides [14,15,17,21], a negative control of water with 50% glycerol (which was subsequently diluted 20× to correspond to the testing condition concentration of venoms), and also three snake venoms from species known to have a generalist diet (*Acanthophis antarcticus*), a fish-specific diet (*Aipysurus laevis*), or a snake-specific diet (*Ophiophagus hannah*). The *B. multicinctus* venom bound to the mimotopes in the assay, thus confirming the validity of the approach (Figure 1), while the negative control showed no binding to the mimotopes (File S1). The dietary test species each showed an expected pattern when tested on the orthosteric sites from diverse target species: the generalist feeder (*A. antarcticus*) did not show phylogenetic bias; the fish specialist (*Aipysurus laevis*) showed a bias towards the fish target; and the snake specialist (*O. hannah*) showed a bias towards the snake target (Figure 1). More specifically, there was only a 46% binding increase between the least (lizard) and the most affected (amphibian) targets for *A. antarcticus*, while the specialized venoms displayed a much greater binding increase between the least and most affected targets, such as for the large increase (340%) between the lizard and snake for *O. hannah*.

These results thus validate the usefulness of our method for determining binding to the orthosteric site of nAChRs of specific taxa, thereby allowing for investigations into prey-specific venom evolution and analyte specificity to these mimotope ligands in general. Previous studies which showed that snake venoms can have prey-specific effects were restricted to the use of model organisms (e.g., domestic chicken to test for bird-specific activity or laboratory mice to test for mammal specific activity). In contrast, our method allows for the testing of activity upon precise prey species, as the orthosteric site sequence can be easily obtained through routine PCR methods, or may already be available on public databases, and the capture peptide synthesized accordingly.

**Figure 2:**
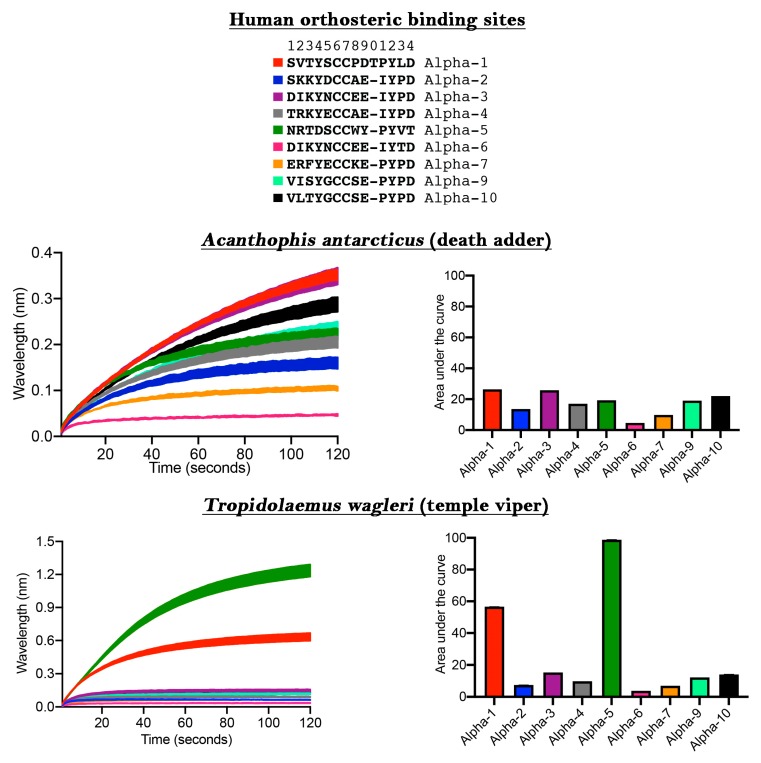
Use of the method for biodiscovery screening for selective ligands of α-nicotinic acetylcholine receptor orthosteric sites. Colored rectangles next to the orthosteric site sequences (top left image) correspond to the results for the particular target in the line and bar graphs. Values are *N* = 3 mean and SEM, with the very small error bars reflective of assay precision.

The ability to discover ligands for specific neurological targets has been limited due to either the difficulty in expressing nAChR within oocytes or the unreliability of receptors expressed on the cell surface in FLIPR assays [13]. We next ascertained the usefulness of our new method for the discovery of novel ligands binding to human nAChRs of therapeutic interest (Figure 2). While the venom of *Acanthophis antarcticus* did not show significant selectivity towards any particular α-nAChR subunit, using this approach we unexpectedly discovered that *Tropidolaemus wagleri* venom contains toxins with a high degree of selectivity for the α-5 subunit. This is an important finding because the α-5 subunit site is of particular interest for the development of anti-smoking medications and colitis [22,23]. Previous studies on neurotoxic peptides from *T. wagleri* venom only examined their interaction with α-1 nAChR [24,25]. Our data, which indicate the presence of molecules in this venom which interact with α-5, demonstrates the power of this new assay to discover future pharmaceuticals and research tools. Specifically, the relevant molecules in *T. wagleri* venom can be used as a probe to deepen our understanding of the subunit structure/function relationship and how this may play a role in the modulation of colitis.

The differential targeting of human receptors is also important in testing venoms for potential clinical effects in the envenomed patient. Previous studies which have relied upon the mollusk acetylcholine binding protein (ACP) used a target that is a very different amino acid sequence in the orthosteric site relative to the human α-1 sequence, including being an amino acid shorter (ACP = SVTYSCCPEAYED compared to human α-1 = SVTYSCCPDTPYLD) or have used a chimera with the human neuronal α-7 at the orthosteric site in the expressed ACP, with human α-7 also differing sharply from human α-1 while also a being a residue shorter (human α-7 = ERFYECCKEPYPD compared to human α-1 = SVTYSCCPDTPYLD) [18,19]. Thus, both approaches use sequences (ACP or α-7) that are bound with less affinity than the venom target (α-1). The limitations of the ACP assay are underscored by some venoms previously well-characterized as neurotoxic (e.g., *Boiga* [26,27,28]) not being active in the ACP assay [18]. 

One of the recent studies above attempted to use ACP with the orthosteric site replaced with the human α-7 to act as a ‘decoy’ molecule (attracting the enzyme away from its intended target via a pseudosubstrate) as a form of novel antivenom [19]. However, as our results strongly suggest (Figure 2), human neuronal α-7 is targeted by venoms at a much lower level than human neuromuscular α-1. In contrast, prior mimotope ‘decoy’ research was based upon the neuromuscular α-1 sequence [14,15,17] and therefore displayed a much greater affinity for venoms than the more recent study using neuronal α-7 [19]. This differential binding is consistent with the venoms being selected for their action on neuromuscular α-1, as this is the only physiologically relevant and likely target reachable by the bloodstream in humans. Therefore, assays based upon the neuronal α-7 subunit target are not valid for ascertaining potential clinical effects or evolutionary patterns due to the lack of real-world relevance combined with the dramatically lower affinity of venoms for α-7 versus α-1. Further, as we have shown taxon-selectivity for α-1, such as fish for the sea snake *Aipysurus laevis* and snakes for *Ophiophagus hannah*, work involving the use of mimotopes as ‘decoys’ with the intent of being a novel antivenom should investigate use of taxon-specific α-1 mimotopes for particular species or basal target species which react broadly yet strongly such as amphibian. 

The proposed use of ACP-based decoy molecules as a therapeutics for snakebite treatment [19] would face several technological and immunological challenges. As ACP is a large, globular protein, it is difficult and expensive to synthetically produce through recombinant expression, which would economically limit its use. In addition, it is a very heat-label protein and thus the use of it as a therapeutic would be limited by the requirement for specialized cold storage. Further, as it is a large foreign protein, it would be very immunogenic and therefore repeated use may result in a violent allergic reaction. In addition to significant limitations in target affinity in the recent work based upon the acetylcholine binding protein and human α-7 chimeras [18,19], the method used in these prior studies also could not provide any binding kinetics data, being able to only separate results into bound or unbound columns. 

In contrast, the mimotope peptides presented in this study are small peptides (14 amino acids) and therefore are both heat stable and also less immunogenic. In contrast to prior work using human α-1 mimotope peptides as decoy molecules [14,15,17], the results in this study show that the affinity for human α-1 may be dramatically lower than that for α-1 from other species. Therefore, mimotope peptides based upon prey lineages may have higher affinity for the toxin molecules, thereby preventing their docking to the pathophysiological target. The method described here may also be useful for investigations of small, linear decoy proteins for potential therapeutic use as antivenom supplements. In addition to target improvements as revealed in this study, our method also provides full kinetics data every 0.2 s, thereby providing a dramatic improvement in understanding the biomolecular interactions.

In summary, our novel method allows for the reliable, high-throughput screening for quantifying the binding kinetics of ligands to nAChRs of any α-subtype from any taxonomical lineage. The high throughput nature of this system combined with the assay set-up we have designed allows for 16 samples to be tested in triplicate in a 45–55-minute period using the Octet RED96 platform. This method enables the examination of evolutionary patterns, the design and testing of new mimotope peptides for use as decoy peptides to supplement antivenom, and for the discovery of novel compounds for drug design and development. This breakthrough in innovation enables for the first time precise measurements of ligand binding to nAChRs spanning the full functional and taxonomical range of potentially affected taxa. As such, our revolutionary assay enables comprehensive characterization of venom/analyte specificity that will shed tremendous light on ecologically, evolutionarily, medically, and economically important analytes and their ligand targets. Our proven technique excelled in the present validation tests investigating both taxon specificity and biodiscovery. Thus, this assay is now validated for use in investigating nAChR binding ligands from multiple perspectives such as potential neurotoxic effects of envenomation on prey and humans and also searching for novel ligands of interest in drug design and development. The flexibility of the approach allows for any potential target to be tested and thus we anticipate that this method will prove to be useful for a broad range of research streams. 

## 3. Materials and Methods 

### 3.1. Venom Collection and Preparation

Venoms were sourced from individual adult snakes (captive and wild-caught) from either the long-term cryogenic collection of the Venom Evolution Laboratory or donated by Venom Supplies Pty Ltd. All venoms were lyophilized and reconstituted in deionized water, centrifuged (4 °C, 5 min at 14,000 RCF), and the supernatant made into a ‘working stock’ (1 mg/mL) with 50% glycerol to prevent freezing at −20 °C where they were stored until use. Protein concentrations were determined in triplicate using a NanoDrop 2000 UV-Vis Spectrophotometer (Thermofisher, Sydney, NSW, Australia) at an absorbance of 280 nm.

### 3.2. Mimotope Production and Preparation

The amino acid sequences for the α-1 orthosteric site for each species were obtained from public databases with the following accession codes: fish α-1 (uniprot P02710), amphibian α-1 (uniprot F6RLA9), lizard α-1 (genbank XM_015426640), avian α-1 (uniprot E1BT92), marsupial α-1 (uniprot G3W0J0), rodent α-1 (uniprot P25108), human α-1 (uniprot G5E9G9), human α-2 (uniprot Q15822), human α-3 (uniprot P32297), human α-4 (uniprot P43681), human α-5 (uniprot P30532), human α-6 (uniprot Q15825), human α-7 (uniprot P36544), human α-9 (uniprot Q9UGM1), and human α-10 (uniprot Q9GZZ6). The only exception was the α-1 sequence for the snake *Coelognathus radiatus*, which was Sanger sequenced by us using the primers (with the M13 primer extension shown in italics): Forward primer sequence – *TGTAAAACGACGGCCAGT*GGAAGCATTTTCCTTTTCAGGAA; Reverse primer sequence-*CAGGAAACAGCTATGAC*GAATGAGAAGAGAAGGCAAGGAAT.

Subsequently, following previous protocols [14,15,16,21] a 13–14 amino acid mimotopes of the acetylcholine orthosteric site of vertebrate α-1 to α-10 nAChRs subunits were synthesized by GenicBio Ltd. (Shanghai, China) based on requested specifications which were adapted from publicly available GenBank sequences and unpublished sequences of cholinergic receptors. As per previous studies [14], the Cys-Cys bridge of the native form was replaced with Ser-Ser for the mimotopes during peptide synthesis steps to avoid uncontrolled postsynthetic thiol oxidation. Research has shown this has no effect on the analyte-ligand complex formation [29,30,31]. The mimotope peptide was then joined to two aminohaxanoic acid (Ahx) spacers to form a 30 Å linker, with the end Ahx then bound to biotin, thereby providing crucial clearance between the biotin and mimotope so that the mimotope maintains its natural conformational freedom when binding to the analyte in solution.

Dried stocks of synthesized mimotopes were solubilized in 100% dimethyl sulfoxide (DMSO) and then diluted 1:10 in deionized water to make a final working stock concentration of 50 µg/mL and stored at −80 °C until use.

### 3.3. Bio-Layer Interferometry (BLI)

Binding kinetics were analyzed by BLI utilizing the Octet Red 96 system (ForteBio). All assays were conducted in standard Greiner black 96 microtiter well plates. Analyte (venom) samples were diluted 1:20 from the working stock to make a final experimental concentration of 50 µg/mL in the well (10 µg per well). Mimotope aliquots were diluted 1:50 to have a final concentration of 1 µg/mL in the well (0.2 µg per well). Assay running buffer was 1X DPBS with 0.1% BSA and 0.05% Tween-20. This buffer inhibits non-specific binding to the surface of the sensor and other proteins. Prior to experimentation, streptavidin sensors were hydrated in the running buffer for 30–60 min, whilst being agitated at 2.0 RPM on a shaker. To regenerate the sensor tips during experimentation, the dissociation of analytes occurs using a standard acidic solution (glycine buffer), made up of 10mM glycine (pH 1.5–1.7) in deionized water.

Octet RED 96 assay methodology in the ForteBio Data Acquisition 9.0 program was set as follows: 60 s baseline, 50 s loading, 120 s baseline, 120 s association, 120 s dissociation, and 80 s regeneration/neutralization step. The regeneration/neutralization step consists of four cycles, lasting 10 s each, alternating between dipping in glycine buffer (regeneration) and then in running buffer (neutralization) per cycle. For each baseline step throughout the experiment the same running buffer was used a maximum of three times per well. Experiments were run at 30 °C with the orbital agitation of the microplate set to 1000 rpm. Experiments were limited to less than 1 h to limit the change in analyte concentration due to evaporation on the warmed plate [32].

Analytes were set up in rows A-H, with triplicates set up in columns 1–3 and 4–6. To account for any potential evaporation effect in the wells during experimentation, the column running order was set to 1, 4, 2, 5, 3, 6 (rather than 1, 2, 3, 4, 5, 6). The mimotopes were set in column 7, running buffer was set in columns 8–10, and regeneration step (glycine buffer) and neutralization step (running buffer) were columns 11 and 12 respectively.

Negative controls consisted of deionized water:glycerol 1:1 mix in replacement of the sample in the wells. *Bungarus multicinctus* venom was used as a positive control, as it has been shown to bind nAChR mimotopes [14,15,16].

### 3.4. Data Processing and Statistical Analysis

Data were processed as follows: 1) raw output folders (one per plate run) containing multiple running files were opened in the ForteBio Data Analysis 9.0 program, and in this program: 2) the ‘sensor tray’ was aligned with the location of sensors on our experimental plate, 3) an inter-step correction was performed whereby the data was aligned to baseline according to the Y-axis of the initial baseline step (0.1–59.9 s), 4) a Savitsky-Golay filter was applied to the data (to remove high-frequency noise from the data), 5) the data were processed according to the above parameters, and 6) exported to Microsoft Excel as a .csv file. The association step data from this .csv file was extracted for each triplicate and imported into Prism 7.0 software (GraphPad Software Inc., La Jolla, CA, USA) where Area Under the Curve (AUC) analyses were conducted and graphs produced.

The phylogenetic trees used were obtained by entering the taxa into timetree.org. These trees were manually recreated using Mesquite software (version 3.2) and then imported to Rstudio (R Core Team, 2015) for all comparative analyses using the APE package for basic data manipulation [33]. In order to investigate the evolutionary relationships of traits, ancestral state reconstructions (ASR) were estimated over the tree using maximum likelihood in the contMap function of the R package phytools [34].

## Supplementary Materials

The following are available online at https://www.mdpi.com/2072-6651/11/10/600/s1, File S1: Water/glycerol negative control mean and standard deviation values.

## References

[1] Fambrough D.M. (1979). Control of acetylcholine receptors in skeletal muscle. Physiol. Rev..

[2] Galzi J.L., Revah F., Bessis A., Changeux J.P. (1991). Functional architecture of the nicotinic acetylcholine receptor: From electric organ to brain. Annu. Rev. Pharmacol. Toxicol..

[3] Gotti C., Clementi F. (2004). Neuronal nicotinic receptors: From structure to pathology. Prog. Neurobiol..

[4] Chambers C., Cutler P., Huang Y.H., Goodchild J.A., Blythe J., Wang C.K., Bigot A., Kaas Q., Craik D.J., Sabbadin D. (2019). Insecticidal spider toxins are high affinity positive allosteric modulators of the nicotinic acetylcholine receptor. FEBS Lett..

[5] Kasheverov I.E., Oparin P.B., Zhmak M.N., Egorova N.S., Ivanov I.A., Gigolaev A.M., Nekrasova O.V., Serebryakova M.V., Kudryavtsev D.S., Prokopev N.A. (2019). Scorpion toxins interact with nicotinic acetylcholine receptors. FEBS Lett..

[6] Schmale D.G., Ault A.P., Saad W., Scott D.T., Westrick J.A. (2019). Perspectives on Harmful Algal Blooms (HABs) and the Cyberbiosecurity of Freshwater Systems. Front. Bioeng. Biotechnol..

[7] Tarvin R.D., Borghese C.M., Sachs W., Santos J.C., Lu Y., O’Connell L.A., Cannatella D.C., Harris R.A., Zakon H.H. (2017). Interacting amino acid replacements allow poison frogs to evolve epibatidine resistance. Science.

[8] Utkin Y., Sunagar K., Jackson T.N.W., Reeks T., Fry B.G., Fry B.G. (2015). Three-Finger Toxins (3FTxs). Venomous Reptiles and Their Toxins: Evolution, Pathophysiology and Biodiscovery.

[9] Debono J., Xie B., Violette A., Fourmy R., Jaeger M., Fry B.G. (2017). Viper Venom Botox: The Molecular Origin and Evolution of the Waglerin Peptides Used in Anti-Wrinkle Skin Cream. J. Mol. Evol..

[10] Fry B.G., Wuster W., Kini R.M., Brusic V., Khan A., Venkataraman D., Rooney A.P. (2003). Molecular evolution and phylogeny of elapid snake venom three-finger toxins. J. Mol. Evol..

[11] Utkin Y.N., Weise C., Kasheverov I.E., Andreeva T.V., Kryukova E.V., Zhmak M.N., Starkov V.G., Hoang N.A., Bertrand D., Ramerstorfer J. (2012). Azemiopsin from *Azemiops feae* viper venom, a novel polypeptide ligand of nicotinic acetylcholine receptor. J. Biol. Chem..

[12] Vulfius C.A., Spirova E.N., Serebryakova M.V., Shelukhina I.V., Kudryavtsev D.S., Kryukova E.V., Starkov V.G., Kopylova N.V., Zhmak M.N., Ivanov I.A. (2016). Peptides from puff adder *Bitis arietans* venom, novel inhibitors of nicotinic acetylcholine receptors. Toxicon.

[13] Fry B.G., Undheim E.A.B., Jackson T.N.W., Roelants K., Georgieva D., Vetter I., Calvete J.J., Scheib H., Cribb B.W., Yang D.C., Fry B.G. (2015). Research Methods. Venomous Reptiles and Their Toxins: Evolution, Pathophysiology and Biodiscovery.

[14] Bracci L., Lozzi L., Lelli B., Pini A., Neri P. (2001). Mimotopes of the nicotinic receptor binding site selected by a combinatorial peptide library. Biochemistry.

[15] Bracci L., Lozzi L., Pini A., Lelli B., Falciani C., Niccolai N., Bernini A., Spreafico A., Soldani P., Neri P. (2002). A branched peptide mimotope of the nicotinic receptor binding site is a potent synthetic antidote against the snake neurotoxin alpha-bungarotoxin. Biochemistry.

[16] Kasher R., Balass M., Scherf T., Fridkin M., Fuchs S., Katchalski-Katzir E. (2001). Design and synthesis of peptides that bind alpha-bungarotoxin with high affinity. Chem. Biol..

[17] Katchalski-Katzir E., Kasher R., Balass M., Scherf T., Harel M., Fridkin M., Sussman J.L., Fuchs S. (2003). Design and synthesis of peptides that bind alpha-bungarotoxin with high affinity and mimic the three-dimensional structure of the binding-site of acetylcholine receptor. Biophys. Chem..

[18] Slagboom J., Otvos R.A., Cardoso F.C., Iyer J., Visser J.C., van Doodewaerd B.R., McCleary R.J.R., Niessen W.M.A., Somsen G.W., Lewis R.J. (2018). Neurotoxicity fingerprinting of venoms using on-line microfluidic AChBP profiling. Toxicon.

[19] Albulescu L.-A., Kazandjian T., Slagboom J., Bruyneel B., Ainsworth S., Alsolaiss J., Wagstaff S.C., Gareth Whiteley G., Harrison R.A., Ulens C. (2019). A decoy-receptor approach using nicotinic acetylcholine receptor mimics reveals their potential as novel therapeutics against neurotoxic snakebite. Front. Pharmacol..

[20] Fortebio Biomolecular Binding Kinetics Assays On The Octet Platform. Https://Www.Moleculardevices.Com/Sites/Default/Files/En/Assets/App-Note/Biologics/Biomolecular-Binding-Kinetics-Assays-On-The-Octet-Platform.Pdf.

[21] Chiappinelli V.A., Weaver W.R., McLane K.E., Conti-Fine B.M., Fiordalisi J.J., Grant G.A. (1996). Binding of native kappa-neurotoxins and site-directed mutants to nicotinic acetylcholine receptors. Toxicon.

[22] Orr-Urtreger A., Kedmi M., Rosner S., Karmeli F., Rachmilewitz D. (2005). Increased severity of experimental colitis in alpha 5 nicotinic acetylcholine receptor subunit-deficient mice. Neuroreport.

[23] Forget B., Scholze P., Langa F., Morel C., Pons S., Mondoloni S., Besson M., Durand-de Cuttoli R., Hay A., Tricoire L. (2018). A human polymorphism in chrna5 is linked to relapse to nicotine seeking in transgenic rats. Curr. Biol..

[24] Molles B.E., Rezai P., Kline E.F., Mcardle J.J., Sine S.M., Taylor P. (2002). Identification Of Residues At The Alpha And Epsilon Subunit Interfaces Mediating Species Selectivity Of Waglerin-1 For Nicotinic Acetylcholine Receptors. J. Biol. Chem..

[25] Molles B.E., Tsigelny I., Nguyen P.D., Gao S.X., Sine S.M., Taylor P. (2002). Residues In The Epsilon Subunit Of The Nicotinic Acetylcholine Receptor Interact To Confer Selectivity Of Waglerin-1 For The Alpha-Epsilon Subunit Interface Site. Biochemistry.

[26] Lumsden N.G., Fry B.G., Kini R.M., Hodgson W.C. (2004). In vitro neuromuscular activity of ‘colubrid’ venoms: Clinical and evolutionary implications. Toxicon.

[27] Lumsden N.G., Fry B.G., Ventura S., Kini R.M., Hodgson W.C. (2004). The in vitro and in vivo pharmacological activity of *Boiga dendrophila* (mangrove catsnake) venom. Autonom. Autacoid Pharmacol..

[28] Lumsden N.G., Fry B.G., Ventura S., Kini R.M., Hodgson W.C. (2005). Pharmacological characterisation of a neurotoxin from the venom of *Boiga dendrophila* (mangrove catsnake). Toxicon.

[29] McLane K.E., Wu X.D., Diethelm B., Conti-Tronconi B.M. (1991). Structural determinants of alpha-bungarotoxin binding to the sequence segment 181-200 of the muscle nicotinic acetylcholine receptor alpha subunit: Effects of cysteine/cystine modification and species-specific amino acid substitutions. Biochemistry.

[30] McLane K.E., Wu X., Conti-Tronconi B.M. (1994). An alpha-bungarotoxin-binding sequence on the *Torpedo* nicotinic acetylcholine receptor alpha-subunit: Conservative amino acid substitutions reveal side-chain specific interactions. Biochemistry.

[31] Tzartos S.J., Remoundos M.S. (1990). Fine localization of the major alpha-bungarotoxin binding site to residues alpha 189–195 of the *Torpedo* acetylcholine receptor. Residues 189, 190, and 195 are indispensable for binding. J. Biol. Chem..

[32] Kamat V., Rafique A. (2017). Designing binding kinetic assay on the bio-layer interferometry (BLI) biosensor to characterize antibody-antigen interactions. Anal. Biochem..

[33] Paradis E., Claude J., Strimmer K. (2004). APE: Analyses of phylogenetics and evolution in R language. Bioinformatics.

[34] Revell L.J. (2012). phytools: An R package for phylogenetic comparative biology (and other things). Method. Ecol. Evolut..

